# Monthly Report of Diseases

**Published:** 1800-08

**Authors:** 


					[ '05 .J
MONTHLY REPORT of DISEASES,
Admitted under the Care of the Physicians of the Finsbv*y
Dispensary* St. John's Square, Clerkenwell.
7'he Distritf, in which the Patients of the Finsbury Dispensary are visited, com-,
prehends the Parishes of St. James, and of St. John, Clcrkenwe/I; of St. Luke*,
tf St. Sepulchre within and without; of St. Bartholomew, the Great and the
Left; the Liberties of the Rolls, and of Glafs?Hctise Yard; the Town of Ifling-
ion; the Parishes 'f St Pancras ; tf St. Andrew, Holborn ; and of St. George
the Martyr, Queen* s-fqU'are. This Trait of Ground may properly enough be term-
ed, a North Western DistriSl of the Metropolis.
v v
List of Diseases, &c. from June 20, to July 20.
No. of Cafes.
Mania ------ 4
Typhus - ----- 29
Pfora - -- -- -- 5
Anafarca ------ 4
Dyfpepfia & Hypochondriacs 21
Amenorrhea - - - 11
Leucorrhcea ----- 6
Menorrhagia - - - - 3
Infantile Difeafes - - - 14
Ccphalasa ----- 3
Dyfentery - - - - - 2
Sore Throat - -, - 1
Hjemorrhois - - - - - 1
Worms - - - - 4
Schrophula - - 6
Jaundice - - - - - 6
No. of Cafe**
Paralyfis - - - - - 2
Cough and Dyfpncea - - 6
Chronic Rheumatiim - - 6
Acute Rheumatifm - - 2
Lumbago - - - - - 1
Diarrhoea ----- 7
Haemoptyfis ----- 3
Pneumonia - - - - - 1
Arthenia ----- 2
Phthifis ------ 8
Hyfteria ------ 3
Petechias - - - <- - 1
Cynanche -- - - - ' - -4.
Pertuflis ------ 2
Herpes ------ 4
Hydrocephalus - - - - 3
The heat of the prefent being fo remarkably more intenfe
than that of many preceding fummers, has prbduced, as might
have been expe&ed, an evident and very important influence
upon the difeafes of the laft month.
Typhus now prevails, attended almoft uniformly with coma,
and a very high degree of delirium.
After the emetic, diluents, and aperients ufually employed
in the firft ftage of this diforder, recourfe was in general had
to the waihing of the patient with cold water,* to the ufe of
the
* In the cafes alluded to there were no conveniences for the adminiftration
?f cold water, in the manner which has b en rtcommended by Dr. Cur-
rie, of Liverpool; whole refpeftable authority would otherwise have been
alone fufficient to have authorifed the experiment. It would l'eem almoft
improper to mention the name of that diltinguilhed writer, without ex-
-preffing an admiration of his talents; and ftill more, of his zealous exer-
tion of them in the advancement of medical information.
? Ht
106 OBservationsDn Dis easts in London.
the Peruvian bark, and, in fome inftances, to a very liberal ad-
miniftratioft of wine; for which, in the cafe of this fever, per -
haps, no adequate fubftitute is to be found amongft all the va-
riety of the Pharmacopeia.
Tbe prefent virulent nature of typhus might be elucidated
a circumftance that is perfonally interefting to the drawef
up of this report. Within the contradled fphere of his know-
ledge in London, more than one medical pra&itioner, in at-
tending patients afHidted with this difeafe, have, during the
courfe of this laft month, fallen a vi&im to the fatal malignity
of its contagion.
One patient, a very fhort time after the attack of the fever,n
was feized with a paroxyfm of madnefs, the violence of which
loon put a final clofe to his exiftence. It fhould be remarked,
however, that in this particular cafe, a ftrong predifpofttion to
infanrty had probably been induced by various habits-of mo-
ral irregularity. When, either by a life of debauchery, or the
corroding operation of any chronic paffion, the ftru&ure of the
mind has been diforganized, there is little hope from either
medical or moral regimen of an entire and permanent reftora-
tion. ? ,
The cafe of mania noticed in the report of laft month as
combined with religious fanaticifm, took place at one of thofe
periods -of life which, in females, operate fo frequently 3s ex-
citing caufes of this difeafe. Another patient, that occurred
about the fame time, became decidedly and violently maniacal,
in confequence, as there was reafon to believe, of a very fevers'
^misfortune which he had experienced on the day preceding
the attack of his diforder. Both of thefe patients were foon
reftored to health, without any conliderable degree of medical
interference. ? .
When mental derangement originates from either of the
fources that gave rife to the complaint in the two instances laft
mentioned i that is, either from a phyfical ftate that exifts only
for a fhort period, or from the fudden impreffion of an unloOk^-
ed-for calamity, an expe?tation of cure may not, perhaps, in
many inftances, be unreafonably entertained.
In the cafes of dyfpepfia and hypochondriafis, which were of
very long {landing, the prefcriptions of the phyficians were
principally confined to country air, cold, and, if poffible,. fea-;
bathing j ^nd, ampngft other things, a refpite from the ufe of
He who affords inftru&ion <0 phyficiaris, mull appear, in an eminent
^free, to deferve the general gratitude of mankind, when it is conhdered
how much their health, a citcumrtance fo eflential to tire value of exigence,
is dependant upon the (kill of that proftflloa. . ,
?- * * drugs.
Observations on Diseases in Ltndon.
IO7
drugs, which, when they become, as in fuch inftances they too
frequently do, the daily food of a pcrfoa for many years, cannot
fail effentially to impair the organs of digeftion, and thereby to
aggravate, in the end, thofe difeafes which they are intended,
and might at firft have been calculated, to relieve. A cafe may
fometimes appear to be the refult of a very long courfe of me-
dicine, merely becaufe a very long courfe of medicine allows
time for the operation of Nature, which of itfelf will' often rer-
ftore a patient, in fpite even of all the naufeating dofes that are
adminiftered. By no means, however, is it intended to dedu&,
in the llighteft degree, from the true value of pharmaceutical
remedies, which are allowed to be, in many inftances, highly
important, and even abfolutely neceflary to the cure of a vaifc
variety of diforders. A proof of their falutary effrcacy was late->
ly exhibited to one of the phyficians of the Difpenfary, in the
tafe of a patient, who, affe&ed with an obftrtnfiion of the bi-
liary du?te, accompanied with the countenance and complexion
appropriate to jaundice, the moft extreme deje&ion of fpirits,
and nearly an entire failure of all the voluntary mufcles, was,
in a few days, reftored to ftrength, cheerfulnefs, and the phy-
fiognomy of health, principally by the energy of mercurial pre-
parations. ' ,
w. w.
Hattcn Garden, July it, 1800. J. R.

				

